# 2732. Peri-Operative Antibiotic Management for Lung Transplant Recipients with Cystic Fibrosis

**DOI:** 10.1093/ofid/ofad500.2343

**Published:** 2023-11-27

**Authors:** Melissa Gordon, Courtney Nichols, Nicholas Marschalk, Sajed Sarwar

**Affiliations:** The Ohio State University Wexner Medical Center, Columbus, Ohio; OSU Wexner Medical Center, Columbus, Ohio; The Ohio State University Medical Center, Columbus, Ohio; The Ohio State University Wexner Medical Center, Columbus, Ohio

## Abstract

**Background:**

Lung transplant (LTx) recipients universally receive peri- and post-transplant antibiotics. Approaches to antibiotics for candidates with cystic fibrosis (CF) are institution specific. Little has been published about these practices. Routine LTx perioperative prophylaxis at our institution is 96 hours of vancomycin and cefepime with antibiotics then adjusted for positive intraoperative donor and recipient cultures. In recipients with CF, perioperative prophylaxis is determined pre-LTx to cover colonizing organisms isolated in culture from the 2 years before LTx, adjusted for intraoperative cultures, and administered for 14-21 days post-LTx. Our study aims to add to the current literature by describing our center’s experience with antibiotic management and outcomes in lung transplant recipients with CF to help guide best practices for prophylaxis strategies for this patient population.

**Methods:**

We performed a retrospective chart review of patients with CF who underwent LTx from 3/1/2015-11/18/21 at our center and were colonized pre-LTx with MRSA, *Pseudomonas aeruginosa*, or *Burkholderia cepacia complex*. Peri- and post-operative antibiotic regimens, donor and recipient culture results, changes in antibiotic therapy post-LTx, and duration of antibiotic therapy were documented. Outcomes included rehospitalization, isolation in culture of the prophylaxis-targeted organisms post-LTx, and mortality, within 6 months of LTx.

**Results:**

Nineteen incidents of LTx were included in this study. Pre-LTx colonizing organisms and antibiotics administered are outlined in table 1. Prophylaxis-targeted organisms were infrequently isolated in culture after LTx (table 2), with 7/19 (36.8%) developing post-transplant infection with prior colonizing organisms. Four (21.1%) recipients developed post-LTx *Clostridioides difficile* infection. One (5.3%) recipient died within 6 months post-LTx.

**Figure:**
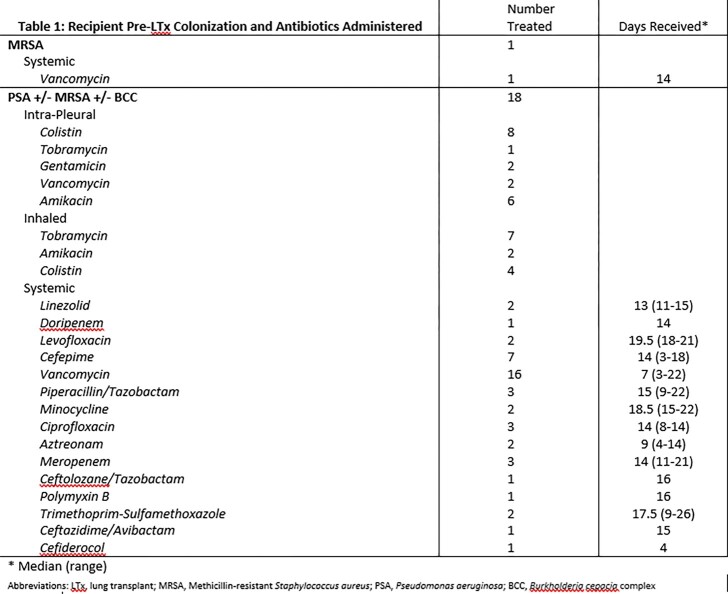

**Figure:**
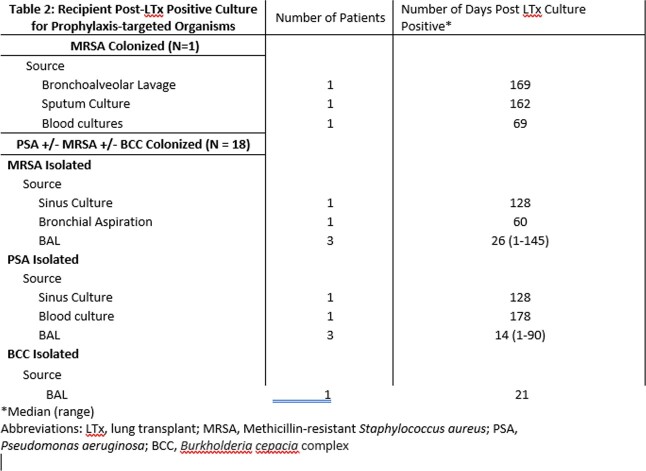

**Conclusion:**

LTx recipients with CF who received targeted perioperative prophylaxis for pre-LTx colonizing organisms infrequently developed post-LTx culture positivity with the same bacteria including none during the antimicrobial prophylaxis period. Future prospective studies are needed to determine optimal antibiotic duration of peri-LTx antibiotics in this patient population.

**Disclosures:**

**All Authors**: No reported disclosures

